# Exploring the adoption of telemedicine and virtual software for care of outpatients during and after COVID-19 pandemic

**DOI:** 10.1007/s11845-020-02299-z

**Published:** 2020-07-08

**Authors:** Anthony Jnr. Bokolo

**Affiliations:** Department of Computer Science, Norwegian University of Science and Technology, NTNU, NO-7491 Trondheim, Norway

**Keywords:** Coronavirus 2019, COVID-19, Outpatient, Pandemic, Telemedicine adoption, Virtual software platforms

## Abstract

As the novel coronavirus disease 2019 (COVID-19) continues to spread across countries, the need for innovative measures to provide high-quality patient care and manage its spread has become more imperative. Software-based systems such as medical software applications could provide valuable suggestion on health-related information to physicians towards improving quality of life, especially for outpatients (e.g., elderly, immunosuppressed, pregnant women). The use of telemedicine and virtual software offers promising potential in the fight against COVID-19. Accordingly, by means of expedited literature and document review, this paper provides implication on the opportunities, application, and challenges of telemedicine and existing virtual software currently adopted as suitable initiatives for reducing the spread of COVID-19. More importantly, findings present factors that impact adoption of telemedicine. The findings suggest that telemedicine and virtual software are capable of decreasing emergency room visits, safeguarding healthcare resources, and lessening the spread of COVID-19 by remotely treating patients during and after the COVID-19 pandemic.

## Introduction

On March 11, 2020, the World Health Organization (WHO) declared the coronavirus disease 2019 (COVID-19) outbreak as a pandemic, with more than 720,000 cases reported over 203 countries as of March 31, 2020. The novel COVID-19 pandemic has altered our society, economy, and healthcare system [1, 2]. Residents were advised to quarantine themselves, and countries have closed their doors to visitors to protect their citizens [3]. Due to the spread of COVID-19, the need for innovative approaches to be employed in controlling the spread has become more imperative [4]. Additionally, COVID-19 pandemic has placed unprecedented reduction on medical resource and increased risk of occupational exposure for medical practitioners [5]. Although addressing the COVID-19 pandemic is multifaceted, one important method that has not yet been fully explored is to leverage information and communications technology (ICT) to support optimal care delivery while lessening the risk of direct human-to-human exposure [6].

Telemedicine which is the deployment of ICT to deliver healthcare digitally can adopted to limit physical human interaction [4]. Telemedicine use has been increasing during the COVID-19 pandemic, being a tool that reaches patients’ home. In this context, telemedicine and virtual software platforms offer affordable, effective, and attractive option. Thus, they can be utilized to manage the pandemic [7]. While this crisis has presented the medical care delivery system with unparalleled challenges [8], COVID-19 has catalyzed rapid use of ICT such as telemedicine and virtual software platforms to deliver healthcare at a distance [9, 10]. Although, telemedicine and virtual software platforms have been used to greatly monitor patients across different medical facilities, it has had fewer applications in larger setting [11]. In the present COVID-19 pandemic, social distancing and quarantine have been adopted as effective methods to reduce the spread of COVID-19. Also, due to the current stay at home measure put in place by governments, telemedicine and virtual software platforms are suggested to provide health services in this study [12].

Telemedicine could also be employed also within hospital wards (for instance, to help attending nurses in intensive care units treating COVID-19 patients in the rehabilitation programme guided by physiotherapists). Therefore, the objective of this study is to provide guidance for treatment of outpatients to help guide medical practitioners as they provide care during and after the COVID-19 pandemic. This paper provides implications on the opportunities, application, and factors that impact adoption of telemedicine and virtual software platforms to reduce the spread of COVID-19 pandemic. Likewise, findings from this paper provide invaluable recommendations related to the impact of telemedicine and virtual software platforms on flattening the infection curve of COVID-19. The remainder of the paper is structured as follows: “Methodology,” “Findings,” “Discussion and implications,” and, lastly, “Conclusion.”

## Methodology

**Fig. 1:**
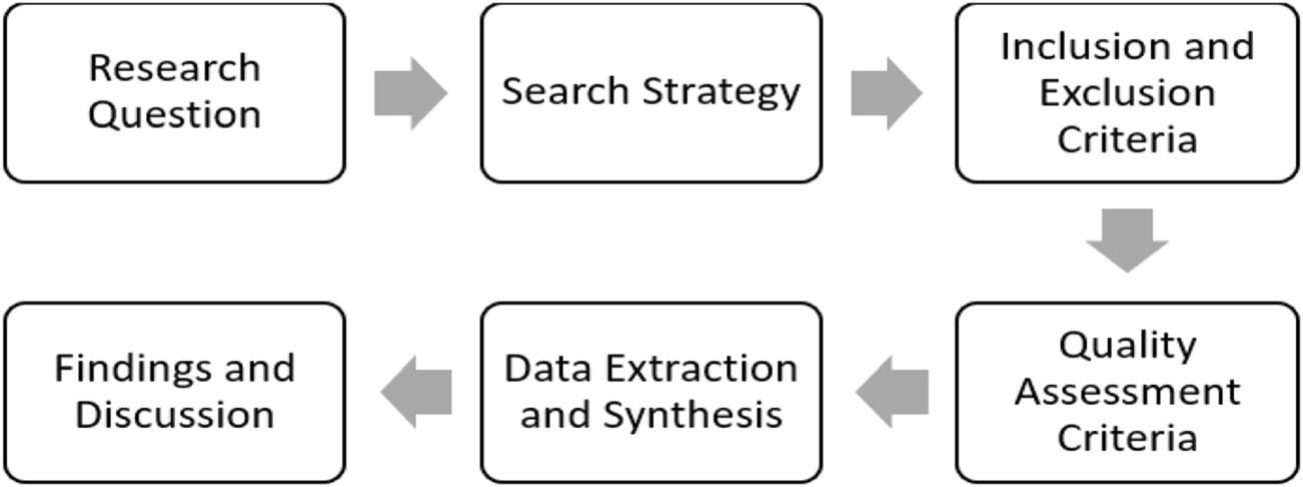
Research design employed in this study

This study adopts an expedited literature review similar to prior study Grimes et al. [13] as a research method based on recommendations from Jnr [14] to guide this study as seen in Fig. 1.

Figure 1 depicts the research design which comprises of six activities: research questions, search strategies, inclusion and exclusion criteria, quality assessment criteria, data extraction and synthesis, and lastly findings and discussion. Each of these activities is briefly discussed below.

### Research questions

Accordingly, this research aims to address the following research questions:

- RQ1: How can telemedicine and virtual software platforms be adopted for treating outpatients during and after COVID-19 pandemic?
- RQ2: What are the telemedicine and virtual software platforms that are adopted during and after COVID-19 pandemic?
- RQ3: What factors impact adoption of telemedicine and virtual software platforms during and after COVID-19 pandemic?

### Search strategy

**Fig. 2:**
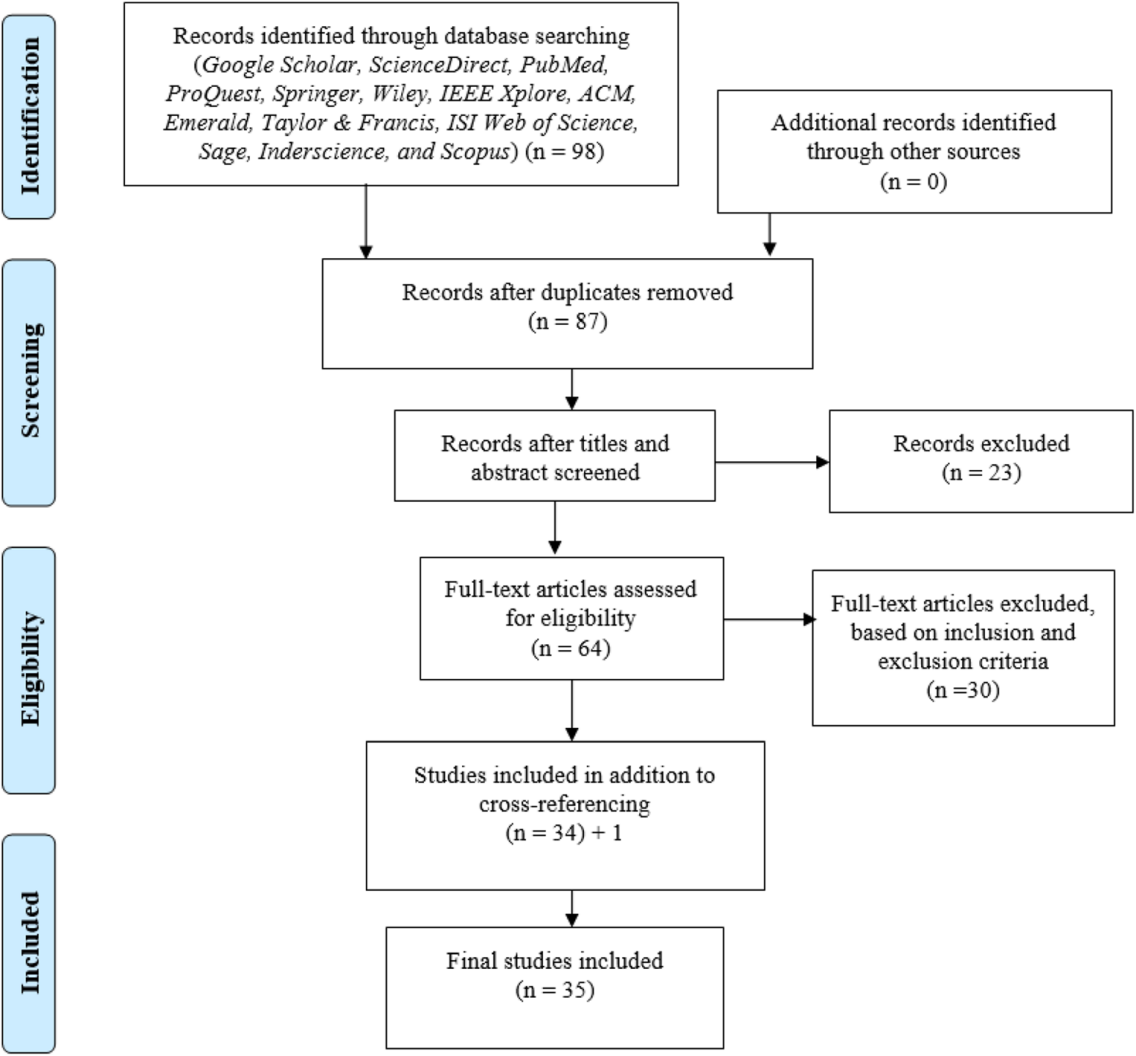
PRISMA flowchart for search process

To extensively search for studies related to telemedicine and virtual software platforms during COVID-19 pandemic, the search strategy was carried out using online databases/libraries. The search was carried out from May 2, 2020 and ended on May 7, 2020 in Google Scholar, ScienceDirect, PubMed, ProQuest, Springer, Wiley, IEEE Xplore, ACM, Emerald, Taylor & Francis, ISI Web of Science, Sage, Inderscience, and Scopus. These online libraries were selected as they are considered appropriate search engines for studies in medical/health science, social science, and information systems research. Additionally, they provide options of carrying out advanced search filtering by keywords and by publication year, type, and research area. Figure 2 depicts the Preferred Reporting Items for Systematic Reviews and Meta-Analysis (PRISMA) flowchart which was employed to search and select papers.

Using these online databases, studies related to telemedicine and virtual software platforms during COVID-19 pandemic were collected from document reports, journal papers, and conference proceedings. Furthermore, in executing the search, specifying keywords or search terms was used to search online electronic databases. To confirm review quality, search keywords were formulated by using Boolean AND/OR operators to combine the search terms to improve the relevance of the search procedure. The main search terms compose of “telemedicine,” “telehealth,” “virtual software platforms,” “digital health,” “virtual medicine,” “e-health,” “COVID-19,” “corona virus 2019,” and “pandemic.” Moreover, a list of search strings which included “digital care, health application, medical application, and e-medicine” was used to search for relevant papers.

As seen in Fig. 2, the search results retrieved 98 articles using the abovementioned keywords. Eleven papers were found as duplicates and were removed. Hence, the total number of remaining papers becomes 87. Next, 23 studies were removed based on title and abstract check assessing if the papers were aligned to the research questions being explored (see “Research questions” section) resulting to 64 papers. The remaining papers were evaluated against the inclusion and exclusion criteria (see “Inclusion and exclusion criteria” section). Therefore, 34 articles were found to meet the inclusion criteria. After which 1 paper was added based on cross-referencing resulting to 35 papers.

### Inclusion and exclusion criteria

After retrieving the papers from the online libraries, each paper was examined based on the inclusion and exclusion criteria as seen in Table 1.

**Table 1:** Inclusion and exclusion criteria

Inclusion criteria	Exclusion criteria
Studies published in English language.	Studies that are not written in English language.
Journal articles, conference proceedings, and document reports.	Not journal articles, conference proceedings, and document reports.
Published between 2020 till date.	Published before 2020.
Studies that provide possible answers to research questions based on title and abstract content.	Remove duplicate/similar studies by retaining the most current and comprehensive version.
Conceptual, literature review, quantitative, qualitative, and experimental studies that provide evidence.	Studies that do not provide any theoretical, empirical, or statistical evidence.
Studies generally related to virtual software platforms and COVID-19 pandemic.	Studies not related to virtual software platforms and COVID-19 pandemic.
Studies related to related to role of ICT to manage COVID-19 pandemic.	Studies not related to role of ICT to manage COVID-19 pandemic.

### Quality assessment criteria

One of the important criteria that is required to be checked along with the inclusion and exclusion criteria is the quality assessment. A higher level of rigorousness of studies is employed to check quality of papers. Thus, a quality assessment checklist confirmed that more than 50% of the selected papers are indexed in ISI Web of Science or Scopus database.

### Data extraction and synthesis

This stage of the review aims to synthesize and categorize the selected papers based on their scope as related to telemedicine and virtual software platforms during COVID-19 pandemic. Thus, the selected studies were reviewed in detail and relevant data were extracted, analyzed, and synthesized to provide answers to the research questions (see “Research questions” section).

## Findings

### Opportunities of adopting telemedicine and virtual software platforms

Several approaches have been considered by different countries to manage and control COVID-19 pandemic. Telemedicine and virtual software platforms are one of the methods to manage COVID-19 pandemic [15, 16]. Given the high risk of transmission of the virus through person-to-person contact, telemedicine can be beneficial in reducing direct contact and helps for patient follow-up [17]. Telemedicine and virtual software platforms overcome physical barriers to provide physicians and patients access to medical care [1]. Adoption of virtual software platforms with telemedicine sustains the continuity of outpatient care during and beyond the COVID-19 pandemic in the midst of social distancing measures, quarantine, and stay at home orders while reducing spread of the virus [5, 13].

Telemedicine also proves useful, in particular, to help conserve personal protective equipment and provide isolated patients connection to friends and family [18]. Accordingly, few medical centers have resorted adopting virtual software platforms such as Microsoft Teams, Zoom, Google Hangouts, Skype, Facebook Messenger, Apple Facetime, and others to facilitate telemedicine care during the pandemic [1]. Respectively, according to Chauhan et al. [6], Doshi et al. [15], and Vidal-Alaball et al. [16], adoption of telemedicine and virtual software platforms aids in the following;

- Decrease the time required to get a diagnosis and initiate treatment, stabilize, or quarantine a patient
- Facilitates close follow-up with patients who can be monitored from their home to avoid oversaturation of health facilities
- Reducing movement of people, minimizing the risk of intra-hospital infection
- Supports co-ordination of medical resources utilized in distant locations
- Prevent the risk of contagion, particularly for medical practitioners, who are key assets
- Aids in informing the general public
- Saves costs on disposable robes, antiseptic material, gloves, disinfecting of hospital spaces, etc.
- Training of medical practitioners who are new to the treatment of pandemic
- Monitoring of real-world data, for example, Johns Hopkins COVID-19 website (https://coronavirus.jhu.edu/map.html) and the VG live corona website update (https://www.vg.no/spesial/2020/corona/) which provide regularly updated information on COVID-19

### Adoption of telemedicine and virtual software platforms for treating outpatients

**Fig. 3:**
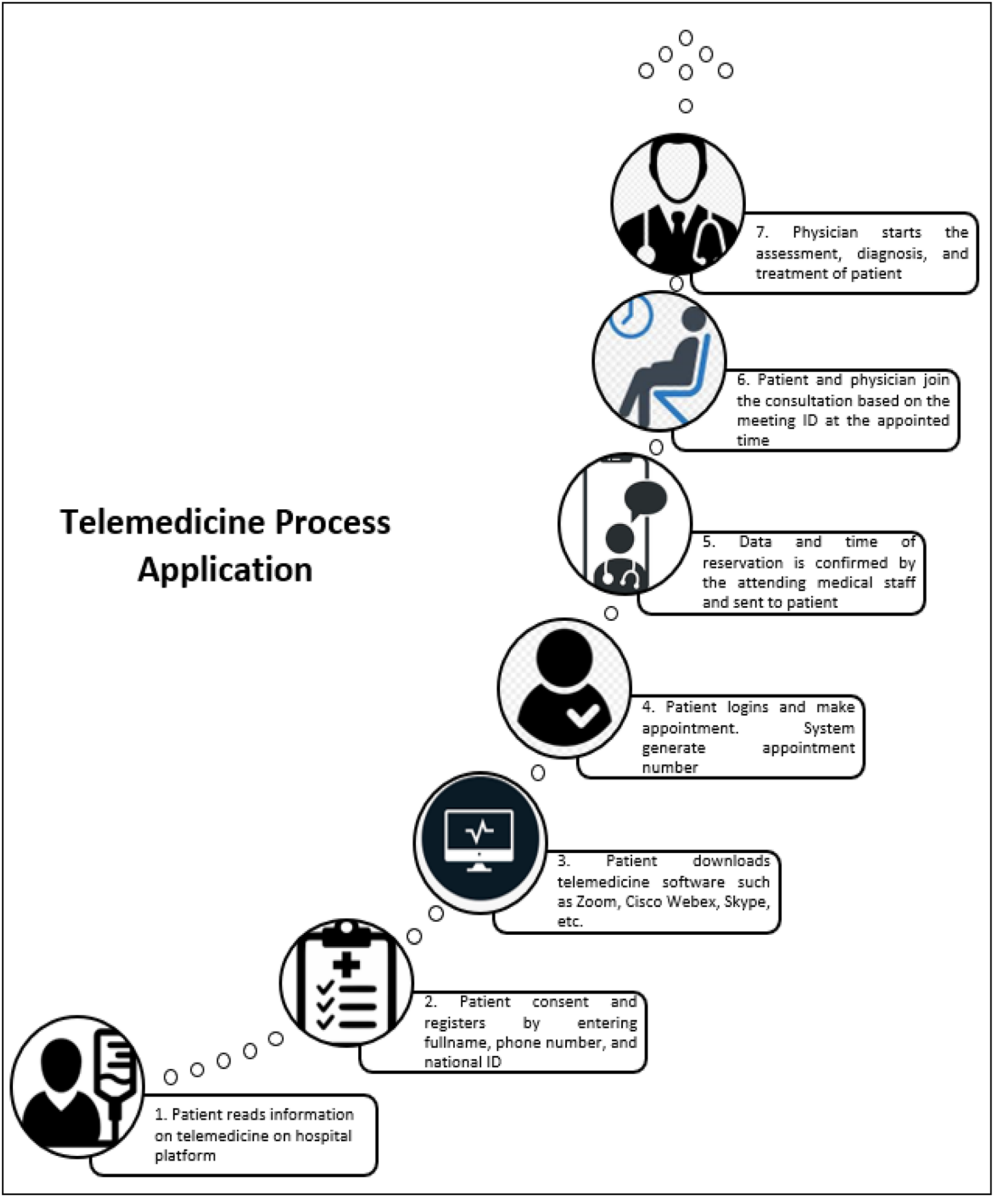
Telemedicine process application

Telemedicine and virtual software platforms have been previously adopted in providing medical care during public health emergencies [5]. Adoption of telemedicine and virtual software platforms provides medical practitioners with reliable information platform that outlines factual information, allowing attending physician to obtain authenticate information in real time [19]. In response to the current COVID-19 pandemic, medical centers are adopting telemedicine and virtual software platforms to provide after hours health coverage from 6 pm to 8 am [5]. An illustration on the application of telemedicine is shown in Fig. 3.

Figure 3 depicts telemedicine process applied for treatment. Thus, telemedicine helps for storage of relevant data, such as physical findings or patient complaints, through transfer of stored video or static images, which can be transferred to the receiving physician to review at a later time [20, 21]. Outpatients can adopt either asynchronous (not same time using e-mail, patient portal messages, health apps, e-consults, etc.) [2, 22, 23] or synchronous (same time using telephone, videoconferencing, etc.) telemedicine and virtual software platforms [10, 11, 18]. As stated by Doshi et al. [15], medical centers are deploying on-site commercial telemedicine carts at medical centers and hospital-provided laptops at home. They also use video software such as Google Duo, Zoom, and Microsoft teams and commercially provided digital stethoscopes and web cameras.

Evidence from Banskota et al. [24], Grimes et al. [13], and Shokri and Lighthall [5] mentioned that patients are happy with the virtual medical experience and appreciate the continuity of obtaining medical care without having to risk their health leaving home during the COVID-19 pandemic. Also, Shokri and Lighthall [5] stated that currently patients are seen in real time using audiovisual appointments, including preliminary consultations, and future follow-ups. However, patients that require urgent medical care are still being examined in person with physicians donning of proper PPE [22]. Thus, telemedicine is not meant to replace in-person healthcare. It allows for protecting patient and physicians from exposure to potential infections and fostering compliance of quarantine and social distancing initiatives [5].

### Telemedicine and virtual software platforms adopted during and after COVID-19 pandemic

As the world faces the impact of COVID-19, several virtual software platforms are being adopted. As recommended by Keesara et al. [25], beyond video consultation software and mobile phone applications, other software platforms such as chatbots and wearable devices are being adopted. These virtual software platforms are deployed to provide asynchronous and synchronous support both for outpatients. Also, these virtual software platforms provide synchronous real-time audiovisual-enabled visits and are easy to use for physicians, patients, and attending staff scheduling appointments, allowing for patient treatment via smart phone computer, or tablet, and conforms to patient privacy laws [5]. Additionally, other emerging virtual software platforms such as voice-interface systems (e.g., Google Voice, Amazon Alexa, Apple Siri) or mobile sensors such as thermometers, smartwatches, or oxygen monitors can be adopted for treatment at home [25].

Although adopting these virtual software platforms in severe situation poses difficulties, many medical-based health systems are already leveraging existing telemedical platforms in their response for treatment of patients [5]. As suggested by Doshi et al. [15], physicians use virtual software platforms, specifically synchronous audio and video applications equipped with high definition videos and digital otoscopes, stethoscopes, dermatoscopes, and ophthalmoscopes used for examining and monitoring patients. In adopting telemedicine and virtual software platforms, physicians must get patient consent for online consultation (mostly automated in virtual software platforms privacy statement seen during installation of the software), document the type of consultation (e.g., synchronous or asynchronous), location of patient and physician, identity confirmation, and documentation of medical service performed (date, time, and duration) [5].

### Factors that impact adoption of telemedicine and virtual software platforms

**Fig. 4:**
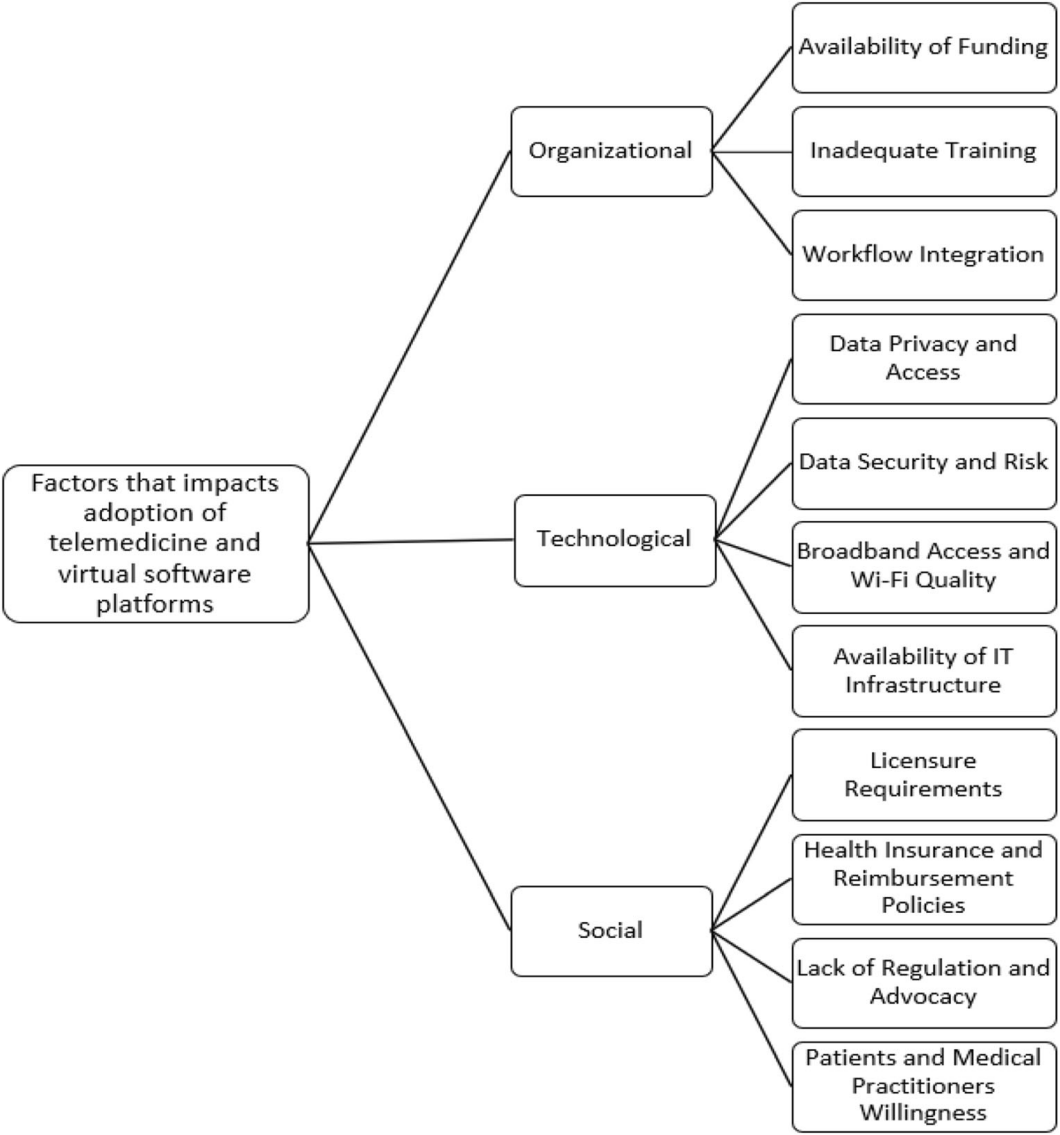
Factors that impacts adoption of telemedicine and virtual software platforms

Telemedicine and virtual software platforms are practically feasible and appropriate for the support of medical practitioners and patients during and after this pandemic via synchronous and asynchronous mediums such as smartphone application, telephone, videoconference, and e-mail [26]. Although the adoption of telemedicine and virtual software platforms holds promise for managing pandemic response, its adoption has limitations such as the availability of robust IT infrastructure, licensing and regulatory services, equipment costs, training of both physicians and nurses, and alterations to integrate within current hospital workflows [3, 15, 27]. Among others, ethical considerations must be taken into account [5]. Accordingly, this study discusses the social, organizational, and technological factors that impact the widespread adoption of telemedicine and virtual software platforms by patients and medical practitioners. The factors derived from the literature are shown in Fig. 4.

Figure 4 depicts the social, organizational, and technological factors that impact the widespread adoption of telemedicine and virtual software platforms by patients and medical practitioners during and after the pandemic. Each of the factors is discussed below.

#### Organizational factors

##### Availability of funding

The deployment of telemedicine and virtual software platforms takes time and does not happen suddenly. It requires funding to purchase resources needed. Also, it involves cost of developing the virtual software platform with expenditures for equipment, medical practitioners’ salaries, information technology (IT) support, and training [15]. As mentioned by Keesara et al. [25], there is need for a broader strategy to cover technical fees that support deployment of needed infrastructures. Thus, the lack of funding is a barrier for adoption of telemedicine [4, 28]. At the moment, a few countries such as the USA, Australia, and China have already invested in telemedicine and are getting promising results. Also, virtual software platforms had been previously adopted by few hospitals in the USA such as University of Pittsburgh, Jefferson Health, and Cleveland Clinic [12]. China has also adopted real-time virtual consultation solution that addresses medical care needs for patients at home [12].

##### Inadequate training

Medical practitioners who interact with patients through virtual care solutions should be trained [5, 29]. Thus, training sessions should be provider and made available when needed [3], either virtually or physically. Similarly, adoption of telemedicine and virtual software platforms might be challenging for some citizens as they will need training in adopting digital technologies [11, 20]. Findings from prior studies [18] suggest that unfamiliarity with virtual software platforms is a key barrier to adoption of telemedicine services [2, 5, 23].

##### Workflow integration

Integrating telemedicine to existing healthcare practice adopted in the hospital that leads to issues on how to manage the use of virtual software platforms for medical treatment of patients by medical practitioners can result to low uptake by both patients and physicians [23]. Thus, workflows for adoption of virtual software platforms should be drafted to minimize burden [30]. Thus, the adopted of telemedicine and virtual software platforms should empower medical practitioners with flexibility in usage for providing medical care [31].

#### Technological factors

##### Data privacy and access

Data privacy and security protection is a critical factor needed for success of telemedicine [32]. The protection and privacy of patient’s data must be important as this has been an issue in the past [11]. Given recent warnings by Federal Bureau of Investigation (FBI) regarding vulnerability of some virtual software platforms, telemedicine must not sacrifice data privacy protections and access measures [18]. But, presently, countries such as China and Singapore have initiated a strict Global Positioning System (GPS) tracking during quarantine raising issues about infringing on citizens liberties and use of private data. Also, adoption of General Data Protection Regulation (GDPR) during and after the pandemic can provide some flexibility as regards to the access and use of citizens’ personal medical data [18]. But for the safety of citizens due to COVID-19, personal medical data of patients could be accessed without the need to obtain their consent. But telemedicine should guarantee data privacy and access protection [16].

##### Data security and risk

The adoption of telemedicine and virtual software platforms involves digital collection and use of sensitive medical information among patients and medical practitioners [5], which could lead to security risk, for the collection, use, and disclosure of sensitive personal data [18]. Findings from recent studies [25, 32] revealed that 94% of participants highlighted data protection regulations as one of the factors that limits adoption of virtual software platforms in health sector. This is true because for instance a mobile medical application may be sponsored by sharing potentially sensitive metadata from health application with third party advertisers that sends ads to patients based on the application used [16].

##### Broadband access and Wi-Fi quality

Studies have shown that the quality of network communication is a key factor that influences adoption of telemedicine. Poor video quality results to decreased engagement and reduced patient satisfaction which affect rapport building among patients and physician [31]. Thus, adequate bandwidth is needed to support the transmission of sound, images, and video data [2]. Consequently, access to good broadband is important for adoption of telemedicine [18]. This factor is mostly a barrier for patients residing in rural areas, which have weak access to the Internet [11]. Thus, it is essential to improve broadband Internet speed to effectively adopt telemedicine for treatment [18, 30].

##### Availability of IT infrastructure

Uncoordinated and poor technology adoption mostly in developing countries is a major barrier to adopting contemporary virtual software platforms and advancements such as telemedicine [4]. This is due to high cost of Internet access, and inadequate IT infrastructure constitutes huge difficulties for adoption of telemedicine in some developing countries [4]. For example, real-time teleconsultation requires uninterrupted communication between patients and physician, hence the availability of audiovisual hardware components with the capability to stream and access high-speed Internet [5].

#### Technological factors

##### Licensure requirements

Stipulated licensure requirements which typically dictate that attending physician must be licensed in the state where the patient is located at the time of service are a barrier to adoption of telemedicine [10]. Hence, given that these state licensures have been barrier to the expansion of telemedicine, it is critical to temporarily suspend restrictions on licensure requirements to adopt telemedicine across state lines [10, 31], to areas of the country that are mostly infected by the pandemic [30, 33]. Thus, licensing policy changes should be established during and after the pandemic without geographical borders or boundaries to physicians [4, 5, 29].

##### Health insurance and reimbursement policies

Currently, most medical insurance does not cover telemedicine treatment and as such do not provide reimbursement for patients. Although, in the USA, all 50 states and the District of Columbia cover some form of fee medical service for live video with Medicaid [33]. Most healthcare insurance coverage such as Medicare has historically had restrictions limiting coverage of telemedicine services to specific geographic areas [33]. But, due to the Coronavirus Preparedness and Response Supplemental Appropriations Act of 2020, geographic restrictions have currently been waived for Medicare reimbursement [10, 15]. Similarly, both the CMS and a few local commercial payers have revised payment policies in response to COVID-19 [3, 13].

##### Lack of regulation and advocacy

As supported from the literature, telemedicine and virtual software platforms can be adopted as an effective tool in helping to manage the current pandemic. However, existing policies are also a barrier that limit how, where, and when telemedicine can be adopted [33]. Findings from a survey conducted by Price Waterhouse Cooper in 2019 revealed that 38% of chief executive officers in US medical care systems reported have no virtual component in their general strategic plan [11, 25]. Moreover, the existence of fewer advocacy bodies such as the physician advocacy groups, the associations for telemedicine, and patient also contributes to the lower adoption of telemedicine and virtual software platforms for treatment [4].

##### Patients’ and medical practitioners’ willingness

The limited adoption of telemedicine and virtual software platforms is mostly attributable to physicians’ unwillingness to adopt telemedicine [24]. Telemedicine is mostly complex and disruptive and thus requires physicians to learn new approach of consulting [28]. Physicians’ acceptance of telemedicine relies on their perception of telemedicine as being normal, safe, and effective. Hence, they may not be familiar and aware of telemedicine, due to limited telemedicine training provided [28]. Likewise, many hospitals are not adopting telemedicine because many patients are not well-versed in virtual software platforms and are initially apprehensive of it leading to resistance and apathy to technological change [4]. Furthermore, in adopting telemedicine and virtual software platforms for treatment, the patient must consent to use of audio and video [13].

## Discussion and implications

The COVID-19 pandemic is rapidly transforming the medical care system, with telemedicine or virtual telemedicine and virtual software platforms being one of the crucial drivers of change [15, 25]. As the health sector continues to be on the front lines, medical centers can leverage on telemedicine to support their patients, protect their staffs, and conserve scarce medical resources [3]. In recent years, growth in telemedicine has been incremental in the world, for example, in the USA, it has been adopted by only 8% as at 2019 [34]. Findings from this study suggest key organizational, technological, and social factors required to improve adoption of telemedicine by patients and medical practitioners.

Additionally, findings from this study depict telemedicine’s transformative impact on healthcare delivery and the rapid shift in virtual software platforms adoption among outpatients and medical practitioners. Thus, telemedicine provides outpatients of all ages access to asynchronous sharing of biometric data via their patient portal and answering pre-set screening question on their mobile devices before audio/video consultation [34]. As pointed out by Mann et al. [34] during the COVID-19 pandemic, outpatients such as pregnant women are routinely synchronizing their home monitoring devices to their physician through electronic health record (HER) and are having advanced post-partum results enabled by telemedicine and virtual software platforms for remote monitoring. This experience may likely create future opportunities of healthcare accessibility and convenience that will be difficult to reverse after the COVID pandemic. Likewise, the regulatory changes raised to support easily accessible adoption of telemedicine may be equally hard to reverse [34].

Evidently, patients and medical practitioners across the world are already adopting telemedicine and virtual software platforms, experiencing their potentials, and establishing expectations and comfort of these software tools for early diagnosis and follow-up [35]. Accordingly, telemedicine and associated virtual software platforms can help to reduce spread of COVID-19 during quarantine and social distancing. Possibly, telemedicine should be adopted as a proactive measure to improve medical care and should not only be seen as a temporary fix in times of emergency; rather, it is a convenient, safe, scalable, effective, and green method of providing medical care [30]. Although, adoption of telemedicine cannot completely solve all the issues caused by COVID-19, it can help flatten the infection curve of COVID-19.

## Conclusion

Telemedicine should be adopted as a proactive measure to improve medical care and should not only be seen as a temporary fix in times of emergency; rather, it is a convenient, safe, scalable, effective, and green method of providing medical care. Therefore, this study explores the significant role of telemedicine and virtual software platforms in managing COVID-19 and provides implications of virtual software platforms during the pandemic outbreak and beyond. Accordingly, by means of expedited literature and document review, this paper discusses the opportunities, application of telemedicine, and existing virtual software adopted as suitable initiatives for reducing the spread of COVID-19. More importantly, findings present the social, technological, and organizational factors that influence the adoption of telemedicine. Findings from this article suggest that technological advances in telemedicine have proven to possess huge potentials in supporting social distancing and quarantine towards controlling the spread COVID-19. The main limitations to this study are aligned to the rapid nature of the review and use of secondary data from existing literature and document reports. Thus, primary data was not employed to validate the social, technological, and organizational factors. Also, the expedited review methods may have inevitably missed some salient studies on COVID-19. Future work will be directed in providing empirical evidence on the identified factors based on primary data from case studies through interview.

## Data Availability

Not applicable.
